# Curcumin suppresses NTHi-induced CXCL5 expression via inhibition of positive IKKβ pathway and up-regulation of negative MKP-1 pathway

**DOI:** 10.1038/srep31695

**Published:** 2016-08-19

**Authors:** Anuhya S. Konduru, Byung-Cheol Lee, Jian-Dong Li

**Affiliations:** 1 Center for Inflammation, Immunity & Infection, Institute for Biomedical Sciences, Georgia State University, Atlanta, GA 30303, USA

## Abstract

Otitis media (OM) is the most common childhood bacterial infection, and leading cause of conductive hearing loss. Nontypeable *Haemophilus influenzae* (NTHi) is a major bacterial pathogen for OM. OM characterized by the presence of overactive inflammatory responses is due to the aberrant production of inflammatory mediators including C-X-C motif chemokine ligand 5 (CXCL5). The molecular mechanism underlying induction of CXCL5 by NTHi is unknown. Here we show that NTHi up-regulates CXCL5 expression by activating IKKβ-IκBα and p38 MAPK pathways via NF-κB nuclear translocation-dependent and -independent mechanism in middle ear epithelial cells. Current therapies for OM are ineffective due to the emergence of antibiotic-resistant NTHi strains and risk of side effects with prolonged use of immunosuppressant drugs. In this study, we show that curcumin, derived from *Curcuma longa* plant, long known for its medicinal properties, inhibited NTHi-induced CXCL5 expression *in vitro* and *in vivo*. Curcumin suppressed CXCL5 expression by direct inhibition of IKKβ phosphorylation, and inhibition of p38 MAPK via induction of negative regulator MKP-1. Thus, identification of curcumin as a potential therapeutic for treating OM is of particular translational significance due to the attractiveness of targeting overactive inflammation without significant adverse effects.

Otitis media (OM) is the most common childhood bacterial infection^1^ with 700 million occurrences globally each year^2^. OM frequently leads to conductive hearing loss, affecting children during the crucial period of speech and language development^3^. The gram-negative bacillus Nontypeable *Haemophilus influenzae* (NTHi) represents the cause of approximately one-third episodes of OM. Current treatments for OM rely on the systemic use of antibiotics, which has led to the emergence of multi-drug resistant bacterial strains^4^,^5^. Therefore, there is an urgent need for developing alternate therapeutic strategies for treating OM.

OM is characterized by the presence of excess inflammation in the middle ear^1^. During infection, epithelial cells act as the first line of defense by secreting numerous pro-inflammatory mediators including chemokines. Chemokines mainly act by recruiting neutrophils to the site of infection. While the appropriate neutrophil response is critical for the removal of the invading pathogen, excess inflammation can lead to tissue damage and perpetuate inflammation leading to detrimental effects, as seen across several inflammatory pathologies including OM^6^,^7^,^8^,^9^. Thus, tight regulation of inflammation is necessary.

CXCL5/ENA-78 (epithelial neutrophil-activating peptide 78) of the Glu-Leu-Arg (ELR) motif-containing C-X-C chemokine family is critical for recruiting neutrophils in response to bacterial infections^10^. Epithelial-cell derived CXCL5 is vital in polymorphonuclear leukocytes (PMN)-driven destructive inflammatory responses in *Mycobacterium tuberculosis*-induced pulmonary tuberculosis. *Cxcl5*^*−/−*^ mice were found to be resistant to fatal tuberculosis^11^. The role of CXCL5 in neutrophil trafficking in lipopolysaccharide (LPS)-induced lung inflammation in mice has been reported^12^. A recent study demonstrated that LPS-induced deregulated CXCL5 expression resulted in exaggerated neutrophil-mediated inflammation in pulmonary bronchiolar cells^13^. CXCL5 is also involved in angiogenesis, tumor growth, and metastasis^14^, with CXCL5 overexpression leading to poor survival in cancer patients^15^. Affymetrix chip analysis on mouse genome revealed marked up-regulation of CXCL5 expression by NTHi in the middle ear of mouse^16^. Middle ear effusion samples from patients with acute and chronic OM have shown the presence of viable NTHi trapped within neutrophil extracellular traps, which continued to elicit inflammatory responses^17^,^18^. Thus, these findings suggest that therapeutic strategies to control aberrant CXCL5 production are of utmost importance for modulating inflammation.

Current anti-inflammatory therapies act via suppressing the positive signaling pathways involved in the production of inflammatory mediators. However, prolonged use of these drugs could have severe side effects because these pathways are also involved in mediating physiological responses. Therefore, therapeutic strategies that increase the levels of endogenous negative regulators of inflammation while leaving the positive pathways intact are gaining prominence^19^. Despite the importance of CXCL5 in mediating inflammation, the molecular mechanisms underlying the up-regulation of CXCL5 production in OM remains largely unknown. Therefore, understanding the mechanism of NTHi-induced CXCL5 regulation in OM will help develop new therapies.

Curcumin, a yellow pigment, common spice derived from the rhizome of *Curcuma longa* plant was long-used for its medicinal properties^20^. Curcumin has been widely reported to have anti-inflammatory, anti-oxidant, anti-microbial, anti-diabetic, anti-carcinogenic anti-tumorigenic, anti-amyloidogenic effects. Due to its tolerability and non-toxicity at high doses, curcumin could be used for prolonged periods without any side effects^20^. Completed clinical trials revealed promising therapeutic effects of curcumin in patients with cancer, inflammatory bowel disease, irritable bowel disease, rheumatoid arthritis, Alzheimer’s disease, and diabetes^21^,^22^,^23^,^24^,^25^,^26^,^27^. More clinical trials evaluating the efficacy of curcumin on a broad range of inflammatory conditions are currently underway. Despite its widely known potent anti-inflammatory properties, the effect of curcumin on NTHi-induced inflammatory responses, especially CXCL5 expression remains to be evaluated.

In the present study, we investigated the underlying molecular mechanism of NTHi-induced CXCL5 expression. We show that NTHi up-regulates CXCL5 expression by activating IKKβ-IκBα and p38 MAPK pathways. Interestingly both pathways mediated CXCL5 expression in an NF-κB-nuclear translocation-dependent and -independent manner, respectively. Also, we show that curcumin suppresses NTHi-induced CXCL5 expression in middle ear epithelial cells. Curcumin suppressed CXCL5 expression by direct inhibition of IKKβ phosphorylation and inhibition of p38 MAPK via induction of negative regulator MKP-1. Thus, our study provides novel insights into the regulation of CXCL5 chemokine and identifies curcumin as a potential therapeutic for treating OM.

## Results

### NTHi induces CXCL5 expression in middle ear epithelial cells *in vitro* and *in vivo*

**Figure 1:**
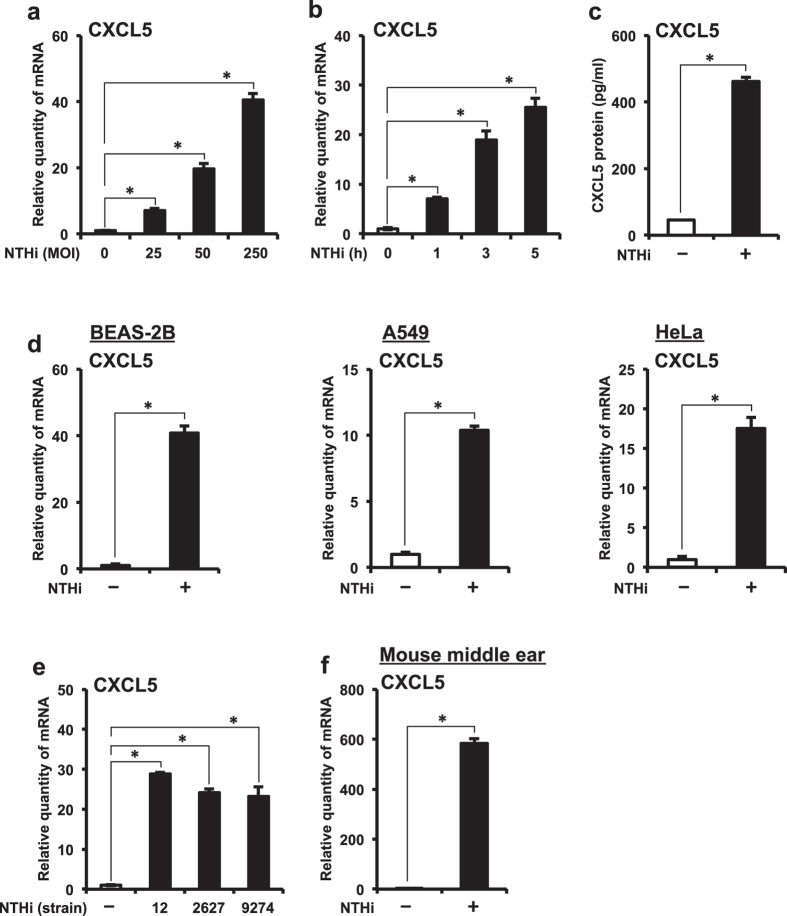
NTHi up-regulates CXCL5 expression in middle ear epithelial cells *in vitro* and *in vivo.* **(a)** HMEECs were stimulated with NTHi (MOI of 25, 50 or 250) for 5 h, and CXCL5 mRNA expression was measured by Q-PCR. **(b)** HMEECs were stimulated with NTHi (MOI of 50) for 1, 3 or 5 h, and CXCL5 mRNA expression was measured by Q-PCR. **(c)** HMEECs were stimulated with NTHi for 12 h, and CXCL5 protein levels in cell culture supernatants was measured by ELISA. **(d)** Airway epithelial BEAS-2B cells, lung epithelial A549 cells and cervical epithelial HeLa cells were stimulated with NTHi for 5 h, and CXCL5 mRNA expression was measured by Q-PCR. **(e)** HMEECs were stimulated with NTHi strains 12, 2627 or 9274 for 5 h, and CXCL5 mRNA expression was measured by Q-PCR. **(f)** Mice were trans-tympanically inoculated with NTHi (6 × 10^7^ CFU) for 6 h, and CXCL5 mRNA expression in middle ear was measured by Q-PCR. Data are mean ± s.d. (n = 3). (**a**,**b,e**) *p < 0.05, ANOVA (Tukey’s post-hoc). (**c**,**d**,**f**) *p < 0.05, t-test. Data are representative of three or more independent experiments.

Epithelial cells act as the first line of defense against injurious stimuli by mediating inflammatory responses. We sought to determine if NTHi induces CXCL5 expression in human middle ear epithelial cells (HMEECs). NTHi induced CXCL5 mRNA expression in a dose- (Fig. 1a) and time-dependent (Fig. 1b) manner in HMEECs. NTHi induced up-regulation of CXCL5 protein expression in HMEECs as quantified by ELISA (Fig. 1c). Effect of NTHi on CXCL5 expression was also confirmed in human airway epithelial BEAS-2B cells, human lung epithelial A549 cells and human cervical epithelial HeLa cells, suggesting the generalizability of this phenomenon to multiple epithelial cells (Fig. 1d). We further explored the generalizability of NTHi-induced CXCL5 expression by employing two additional commonly used clinical NTHi strains 2627 and 9274 known to cause OM^28^,^29^. NTHi strains 2627 and 9274 also induced CXCL5 mRNA expression at levels comparable to that of NTHi strain 12 (used throughout the study) in HMEECs (Fig. 1e). Consistent with the *in vitro* findings, NTHi also induced up-regulation of CXCL5 mRNA in mouse middle ear (Fig. 1f).

### TLR2-MyD88-TRAF6-TAK1 signaling axis is required for NTHi-induced CXCL5 expression

**Figure 2:**
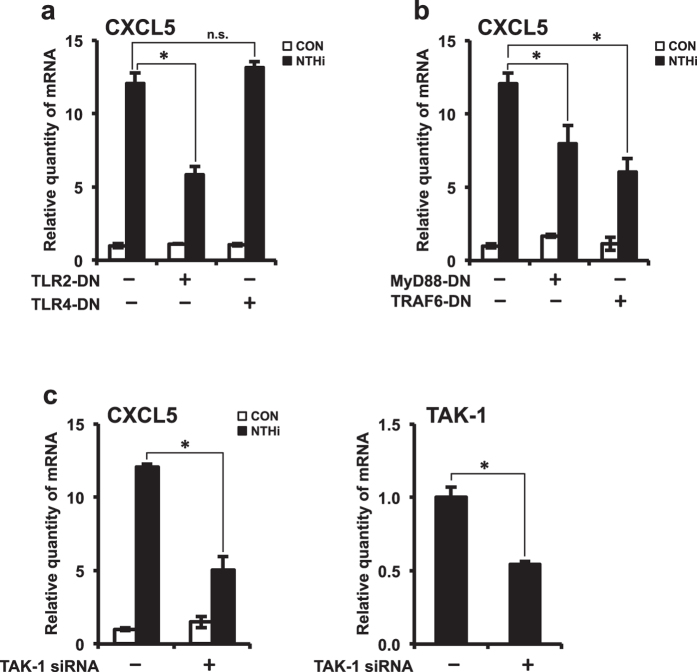
TLR2-MyD88-TRAF6-TAK1 signaling axis is required for NTHi-induced CXCL5 expression. **(a**,**b**) HMEECs were transfected with Mock, TLR2-DN, TLR4-DN, MyD88-DN or TRAF6-DN plasmid. Cells were stimulated with NTHi for 5 h, and CXCL5 mRNA expression was measured. **(c)** HMEECs were transfected with control siRNA or TAK1 siRNA. Cells were stimulated with NTHi for 5 h, and CXCL5 mRNA expression was measured. Knockdown of TAK1 by siRNA was confirmed by Q-PCR. Data are mean ± s.d. (n = 3). (**a**,**b**) *p < 0.05, ANOVA (Tukey’s post-hoc). (**c**) *p < 0.05, t-test. Data are representative of three or more independent experiments.

Toll-like receptors (TLRs) are cell surface receptors that play a critical role in mounting early innate immune responses against invading pathogens. TLRs recognize conserved motifs known as pathogen-associated microbial patterns (PAMPs), expressed on microbial pathogens and initiate signaling cascades leading to the production of pro-inflammatory mediators. To date, at least 11 human TLRs have been identified. Among them, TLR2 is known to recognize lipopolysaccharide (LPS), characteristic of Gram-negative bacteria. Based on our previous finding that TLR2 mediated NTHi-induced pro-inflammatory signaling cascades, we sought to determine its role in CXCL5 chemokine production. HMEECs were transfected with TLR dominant-negative mutants TLR2-DN and TLR4-DN. Overexpression of TLR2-DN significantly decreased NTHi-induced CXCL5 mRNA expression, whereas TLR4-DN had no significant effect on CXCL5 expression (Fig. 2a). Next, we sought to determine the signaling molecules downstream of TLR2, involved in mediating CXCL5 expression. Following recognition of NTHi by TLR2, myeloid differentiation factor 88 (MyD88) adaptor protein is recruited to the receptor. MyD88 then recruits and activates IL-1 receptor-associated kinases (IRAKs), which further lead to the recruitment and activation of tumor-necrosis factor-receptor-associated factor 6 (TRAF6). IRAK-TRAF6 complex dissociates from the receptor bound complex and further interacts with transforming growth factor-β- activated kinase 1 (TAK1). Activation of TAK1 leads to initiation of further downstream signaling pathways, resulting in nuclear translocation of transcription factors, and in turn regulates the expression of pro-inflammatory mediators. To determine the involvement of MyD88 and TRAF6 in CXCL5 expression, HMEECs were transfected with dominant-negative mutants MyD88-DN and TRAF6-DN. Overexpression of MyD88-DN and TRAF6-DN significantly suppressed NTHi-induced CXCL5 expression (Fig. 2b). Depletion of endogenous TAK1 with TAK1 siRNA also decreased CXCL5 mRNA expression (Fig. 2c). TAK1 siRNA knockdown efficiency was confirmed by Q-PCR. Therefore, these results suggest that TLR2-MyD88-TRAF6-TAK1 signaling axis is required for NTHi-induced CXCL5 expression.

### Activation of IKKβ-IκBα signaling pathway is required for NTHi-induced CXCL5 expression

**Figure 3:**
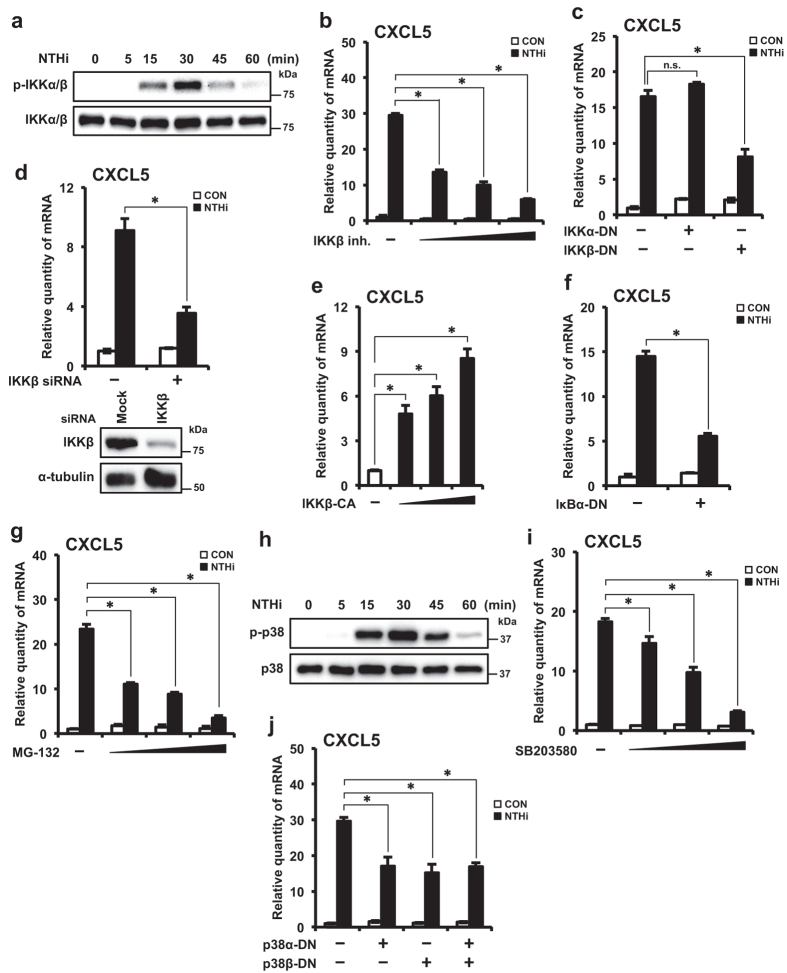
Activation of IKKβ-IκBα and p38 signaling pathways are required for NTHi-induced CXCL5 expression. **(a)** HMEECs were stimulated with NTHi for various time intervals as indicated in the figure. Phospho-IKKα/β, total IKKα/β protein levels were visualized by western blot. **(b)** HMEECs were pre-treated with IKKβ inhibitor (0.25, 0.5 or 1.0 μM) for 1 h, followed by stimulation with NTHi for 5 h, and CXCL5 mRNA expression was measured. **(c)** HMEECs were transfected with Mock, IKKα-DN or IKKβ-DN plasmid. Cells were stimulated with NTHi for 5 h, and CXCL5 mRNA expression was measured. **(d)** HMEECs were transfected with control siRNA or IKKβ siRNA. Cells were stimulated with NTHi for 5 h, and CXCL5 mRNA expression was measured. Knockdown of IKKβ protein by siRNA was confirmed by western blot. **(e)** HMEECs were transfected with Mock, IKKβ–CA (0.25, 0.5 or 1 μg) plasmid. CXCL5 mRNA expression was measured. **(f)** HMEECs were transfected with Mock or IκBα (S32A/S36A) plasmid. Cells were stimulated with NTHi for 5 h, and CXCL5 mRNA expression was measured. **(g)** HMEECs were pre-treated with MG-132 (5, 10 or 20 μM) for 1 h, followed by stimulation with NTHi for 5 h, and CXCL5 mRNA expression was measured. **(h)** HMEECs were stimulated with NTHi for various time intervals as indicated in the figure. Phospho-p38, total p38 protein levels were visualized by western blot. **(i)** HMEECs were pre-treated with SB203580 (5, 10 or 20 μM) for 1 h, followed by stimulation with NTHi for 5 h, and CXCL5 mRNA expression was measured. **(j)** HMEECs were transfected with Mock, p38α–DN, p38β–DN or both (p38α–DN and p38β–DN) plasmids. Cells were stimulated with NTHi for 5 h, and CXCL5 mRNA expression was measured. Data are mean ± s.d. (n = 3). (**b**,**c**,**e,g,i,j)** *p < 0.05, ANOVA (Tukey’s post-hoc). (**d,f**) *p < 0.05, t-test. n.s., not significant. Displayed immunoblots are cropped images from full-length blots, presented in Supplementary Fig. S1. Data are representative of three or more independent experiments.

Previous studies have shown that IKKβ signaling axis is crucial for NTHi-induced inflammatory responses^30^. Therefore, we examined the role of IKKβ in up-regulation of CXCL5. We first confirmed that NTHi activates IKKβ in HMEECs. IKKα/β phosphorylation was observed at 15 minutes, followed by a peak at 30 minutes, and declined after that (Fig. 3a). To determine if IKKβ is required for NTHi-induced CXCL5 expression, we used multiple approaches. IKKβ inhibitor significantly suppressed NTHi-induced CXCL5 mRNA expression in a dose-dependent manner (Fig. 3b). To identify the major IKK isoform involved in NTHi-induced CXCL5 regulation, HMEECs were transfected with IKK dominant-negative (DN) mutants IKKα-DN and IKKβ-DN. Overexpression of IKKβ-DN significantly decreased NTHi-induced CXCL5 mRNA expression, whereas IKKα-DN had no significant effect (Fig. 3c). Consistent with this result, depletion of endogenous IKKβ with IKKβ siRNA also decreased CXCL5 mRNA expression (Fig. 3d). IKKβ siRNA knockdown efficiency was confirmed by Western blot. To further confirm that activated IKKβ induces CXCL5 expression, HMEECs were transfected with constitutively active (CA) form IKKβ-CA. Overexpression of IKKβ-CA markedly induced CXCL5 expression in a dose-dependent manner (Fig. 3e). Phosphorylation and proteasomal degradation of IκBα, by IKKβ, are required for signal^31^. Overexpression of a trans-dominant mutant form of IκBα (IκBα-DN) suppressed NTHi-induced CXCL5 expression (Fig. 3f). To further confirm that IκBα degradation is essential for NTHi-induced CXCL5 up-regulation, we used MG-132 proteasome inhibitor. MG-132 significantly suppressed NTHi-induced CXCL5 mRNA expression in a dose-dependent manner (Fig. 3g). Thus, these results suggest that IKKβ-IκBα signaling pathway is required for CXCL5 induction by NTHi.

### Activation of p38 signaling is required for NTHi-induced CXCL5 expression

NTHi has been shown to mediate inflammatory responses via activation of p38 MAPK signaling axis in addition to activation of IKKβ-IκBα pathway^31^. Therefore, we examined the role of p38 in up-regulation of CXCL5. We first confirmed that NTHi activates p38 MAPK in HMEECs. p38 phosphorylation was observed at 15 minutes, followed by a peak at 30 minutes, and declined after that (Fig. 3h). To determine if p38 MAPK activation is essential for NTHi-induced CXCL5 expression, multiple approaches were used. SB203580, a specific inhibitor of p38 activation, significantly suppressed NTHi-induced CXCL5 mRNA expression in a dose-dependent manner (Fig. 3i). To identify the major p38 isoform involved in NTHi-induced CXCL5 regulation, HMEECs were transfected with p38 DN mutants p38α-DN and p38β-DN. Over-expression of either or both p38α, p38β DN forms significantly decreased NTHi-induced CXCL5 expression (Fig. 3j), consistent with SB203580 data. Together, these data suggest that p38 pathway is required for induction of CXCL5.

### IKKβ-IκBα and p38 signaling axes mediate CXCL5 induction via p65 nuclear translocation-dependent and -independent mechanism, respectively

**Figure 4:**
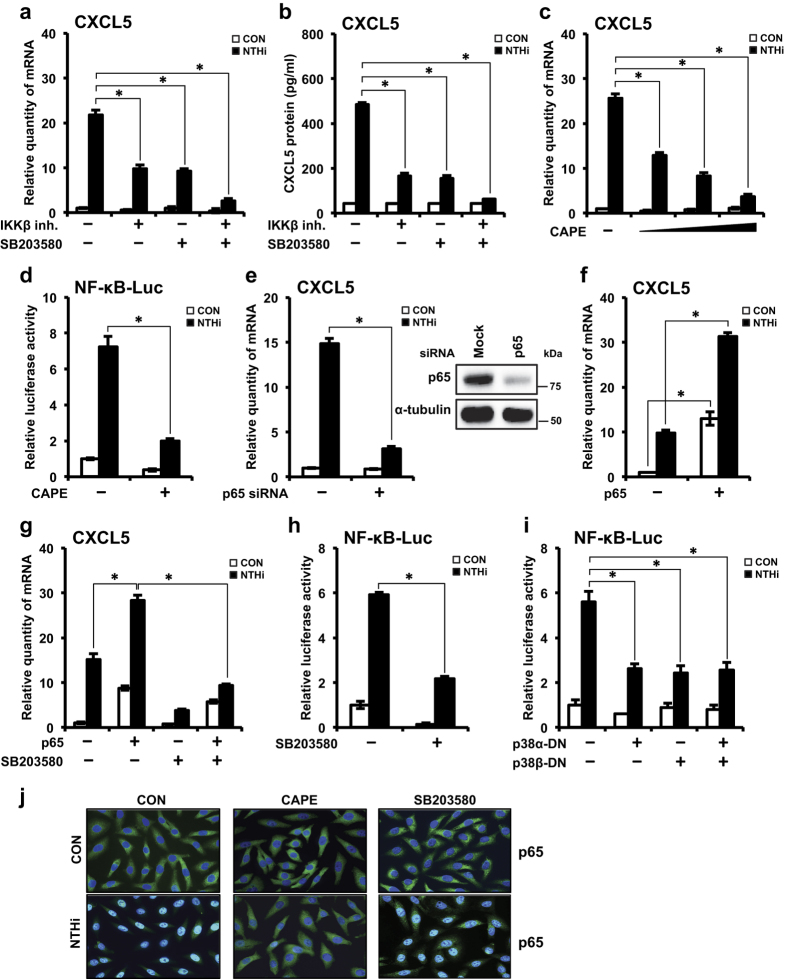
IKKβ-IκBα and p38 signaling axes mediate CXCL5 induction via p65 nuclear translocation–dependent and –independent mechanism, respectively. (**a**,**b**) HMEECs were treated with IKKβ inhibitor (0.5 μM), SB203580 (10 μM) or both for 1 h, followed by stimulation with NTHi for *(a)* 5 h, and CXCL5 mRNA expression was measured, *(b)* 12 h, and CXCL5 protein levels in cell culture supernatants was measured by ELISA. (**c**) HMEECs were pre-treated with CAPE (5, 10 or 25 μg/ml) for 1 h, followed by stimulation with NTHi for 5 h, and CXCL5 mRNA expression was measured. (**d**) HMEECs were transfected with NF-κB luciferase vector. Cells were pre-treated with CAPE (25 μg/ml) for 1 h, followed by NTHi stimulation for 5 h. NF-κB promoter activity was measured by luciferase assay. (**e**) HMEECs were transfected with control siRNA or p65 siRNA. Cells were stimulated with NTHi for 5 h, and CXCL5 mRNA expression was measured. Knockdown of p65 protein by siRNA was confirmed by western blot. (**f**,**g**) HMEECs were transfected with Mock or p65. Cells were *(f)* stimulated with NTHi for 5 h, *(g)* pre-treated with SB203580 (20 μM) for 1 h, followed by stimulation with NTHi for 5 h; and CXCL5 mRNA expression was measured. (**h**,**i**) HMEECs were transfected with *(h)* NF-κB luciferase vector alone, *(i)* NF-κB luciferase vector and Mock, p38α–DN, p38β–DN or both (p38α–DN and p38β–DN) plasmids. Cells were *(h)* pre-treated with SB203580 (20 μM) for 1 h and *(h,i)* stimulated with NTHi for 5 h. NF-κB promoter activity was measured by luciferase assay. (**j**) HMEECs were pre-treated with CAPE (25 μg/ml) or SB203580 (20 μM) for 1 h, followed by NTHi stimulation for 1 h. p65 translocation was visualized by immunofluorescence by FITC staining. DAPI, nuclear stain. Magnification: 400x. Data are mean ± s.d. (n = 3). (**a**–**c**,**f**,**g**,**i**) *p < 0.05, ANOVA (Tukey’s post-hoc). (**d**,**e**,**h**) *p < 0.05, t-test. Displayed immunoblots are cropped images from full-length blots, presented in Supplementary Fig. S2. Data are representative of three or more independent experiments.

Next, we sought to determine how IKKβ-IκBα and p38 signaling pathways induce CXCL5 expression. Co-treatment with IKKβ inhibitor and SB203580 synergistically suppressed CXCL5 expression at mRNA (Fig. 4a) and protein (Fig. 4b) levels. As NF-κB is a known major transcription factor for pro-inflammatory mediators^32^, we investigated its involvement in NTHi-induced CXCL5 expression. Nuclear translocation of NF-κB is critical for its activity^32^. NTHi induces nuclear translocation of p65, the major subunit of NF-κB complex^31^. To determine that p65 nuclear translocation and activation is required for NTHi-induced CXCL5 expression, we used multiple approaches. Pretreatment with caffeic acid phenyl ester (CAPE), a specific inhibitor of NF-κB nuclear translocation (independent of IκBα degradation), completely abrogated NTHi-induced CXCL5 expression (Fig. 4c). Pretreatment with CAPE markedly diminished NTHi-induced NF-κB promoter-driven luciferase activity (Fig. 4d). Depletion of endogenous p65, with p65 siRNA, decreased CXCL5 mRNA expression (Fig. 4e). p65 siRNA knockdown efficiency was confirmed by Western blot. Overexpression of p65 further enhanced NTHi-induced CXCL5 expression (Fig. 4f). Therefore, these results suggest that NTHi induces CXCL5 transcription via activation of NF-κB, specifically by p65 subunit.

To determine whether p38 MAPK induces CXCL5 expression via p65, HMEECs transfected with p65 were pre-treated with SB203580, prior to NTHi stimulation. SB203580 decreased CXCL5 expression in p65-transfected cells (Fig. 4g). We further confirmed the requirement of p38 for NF-κB activation by multiple approaches. Pre-treatment with SB203580 markedly decreased NTHi-induced NF-κB promoter-driven luciferase activity (Fig. 4h). Consistent with this result, over-expression of either or both p38α and p38β DN forms decreased NTHi-induced NF-κB promoter activity (Fig. 4i). These results suggest that p38 MAPK also mediates NTHi-induced CXCL5 expression in a p65 dependent mechanism.

Since nuclear translocation of p65 is imperative for NF-κB-driven gene expression^32^, we determined if p38 up-regulates CXCL5 expression via facilitating nuclear translocation of p65 by immunofluorescence staining. CAPE markedly inhibited nuclear translocation of p65, whereas SB203580 did not show any significant effect (Fig. 4j). Taken together these results suggest that p38 mediates CXCL5 expression via a mechanism independent of the nuclear translocation of p65. Therefore, these results suggest that IKKβ and p38 signaling pathways mediate NTHi-induced CXCL5 up-regulation via activation of p65 in a p65-nuclear translocation-dependent and -independent mechanism, respectively.

### Curcumin suppresses NTHi-induced CXCL5 expression *in vitro* and *in vivo*

**Figure 5:**
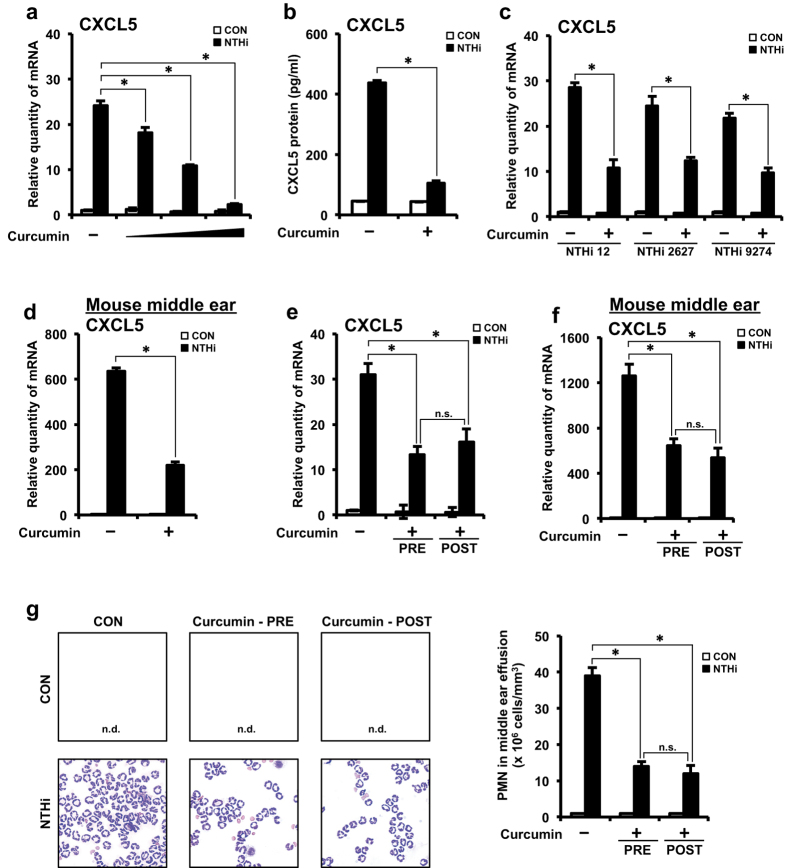
Curcumin suppresses NTHi-induced CXCL5 expression *in vitro* and *in vivo*. **(a)** HMEECs were pre-treated with curcumin (10, 20 or 50 μM) for 1 h, followed by stimulation with NTHi for 5 h, and CXCL5 mRNA expression was measured. **(b)** HMEECs were pre-treated with curcumin (20 μM) for 1 h, followed by stimulation with NTHi for 12 h, and CXCL5 protein levels in cell culture supernatants was measured by ELISA. **(c)** HMEECs were pre-treated with curcumin (20 μM) for 1 h, followed by stimulation with NTHi strains 12, 2627 or 9274 for 5 h, and CXCL5 mRNA expression was measured. **(d)** Mice were pretreated with curcumin (50 mg/kg) (i.p) for 1 h, followed by trans-tympanic inoculation with NTHi (5 × 10^7^ CFU) for 6 h, CXCL5 mRNA expression in dissected middle ear was measured. **(e)** HMEECs were pre-treated with curcumin (20 μM) 1 h prior NTHi stimulation or post-treated with curcumin (20 μM) 1 h after NTHi stimulation. 5 h after NTHi stimulation CXCL5 mRNA expression was measured. **(f)** Mice were pre-treated with curcumin (50 mg/kg) (i.p) for 1 h, followed by trans-tympanic inoculation with NTHi or post-treated with curcumin (50 mg/kg) (i.p) 1 h after NTHi inoculation. 6 h after NTHi inoculation CXCL5 mRNA expression in dissected middle ear was measured. **(g)** Mice were pre-treated with curcumin (50 mg/kg) (i.p) for 1 h, followed by trans-tympanic inoculation with NTHi or post-treated with curcumin (50 mg/kg) (i.p) 1 h after NTHi inoculation. Middle ear effusion was harvested 9 h after NTHi inoculation. Following cytocentrifugation, cells were stained with Diff-Quik staining kit. n.d., not detected. Magnification: 400x. PMN cell count in middle ear effusion was determined using a hemocytometer under the microscope. Data are mean ± s.d. (n = 3). (**a**,**e–g)** *p < 0.05, ANOVA (Tukey’s post-hoc). (**b–d**) *p < 0.05, t-test. n.s., not significant. n.d., not detected. Data are representative of three or more independent experiments.

Having identified the molecular mechanisms underlying NTHi-induced up-regulation of CXCL5, we next sought to explore the translational significance of these findings. Because curcumin, a promising anti-inflammatory agent, has previously been shown to inhibit NF-κB^33^,^34^, we first determined if curcumin inhibits NTHi-induced up-regulation of CXCL5. Curcumin pre-treatment inhibited CXCL5 mRNA expression in a dose-dependent manner (Fig. 5a). Curcumin’s inhibitory effect on CXCL5 protein levels was also confirmed (Fig. 5b). Additionally, the inhibitory effect of curcumin on CXCL5 mRNA expression was also observed in HMEECs stimulated with other common clinical NTHi strains 2627 and 9274 (Fig. 5c), thereby suggesting the generalizability to more OM-causing NTHi strains. Consistent with *in vitro* findings, curcumin pre-treatment inhibited CXCL5 mRNA expression in the middle ear of mice inoculated with NTHi (Fig. 5d). These data suggest that curcumin inhibits NTHi-induced CXCL5 expression in middle ear epithelial cells *in vitro* and *in vivo*.

Next, we sought to determine the therapeutic relevance of the inhibitory effect of curcumin in NTHi-induced OM model. Thus, we evaluated the effect of administering curcumin post-NTHi infection that resembles a clinically relevant setting. Administering curcumin post-NTHi infection significantly suppressed NTHi-induced CXCL5 mRNA expression *in vitro* (Fig. 5e) and *in vivo* (Fig. 5f). Curcumin suppressed CXCL5 expression to the same extent under both pre-NTHi and post-NTHi infection conditions. Since CXCL5 is a neutrophil chemoattractant, we further evaluated the effect of curcumin on PMN recruitment in response to NTHi infection in a mouse model of OM. Consistent with the above results curcumin (pre-NTHi and post-NTHi infection) inhibited PMN infiltration as assessed by PMN staining of middle ear effusion from mice (Fig. 5g). Thus, these data suggest that curcumin is a potential therapeutic for treating NTHi-induced inflammation as seen in OM.

### Curcumin suppresses NTHi-induced CXCL5 expression via inhibition of IKKβ and p38 pathways

**Figure 6:**
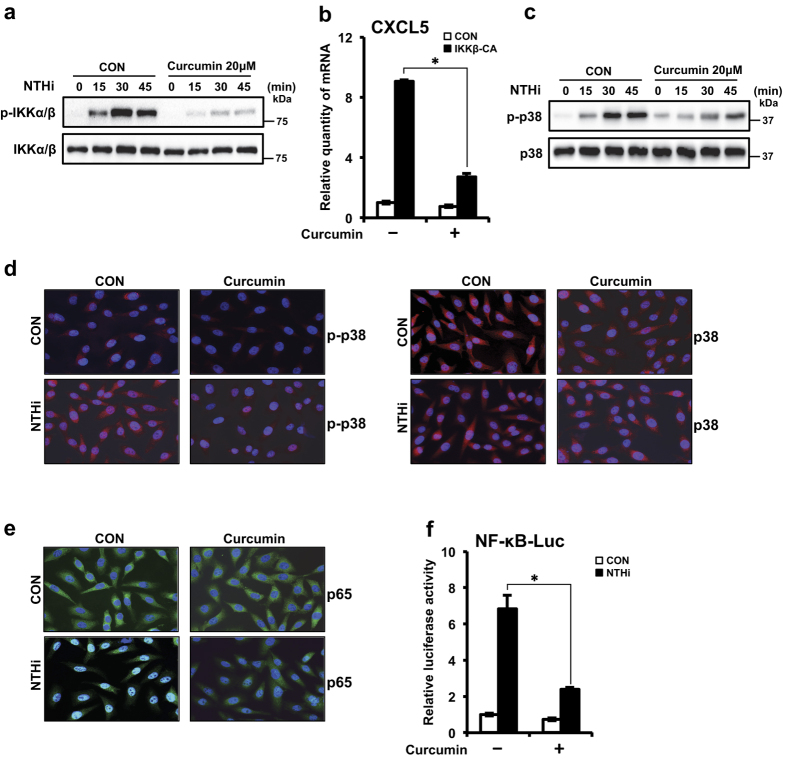
Curcumin suppresses NTHi-induced CXCL5 expression via inhibition of IKKβ and p38 pathways. **(a)** HMEECs were treated with curcumin (20 μM) for 1 h, followed by stimulation with NTHi for various time intervals as indicated in the figure. Phospho-IKKβ, total IKKβ protein levels were visualized by western blot. **(b)** HMEECs were transfected with Mock or IKKβ–CA plasmid. Cells were treated with curcumin (20 μM) for 1 h, and CXCL5 mRNA expression was measured. **(c)** HMEECs were treated with curcumin (20 μM) for 1 h, followed by stimulation with NTHi for various time intervals as indicated in the figure. Phospho-p38, total p38 protein levels were visualized by western blot. **(d**,**e)** HMEECs were pre-treated with curcumin (20 μM) for 1 h, followed by NTHi stimulation for *(d)* 30 min, *(e)* 1 h. *(d)* Phospho-p38, total p38 protein levels (Rhodamine stain), *(e)* p65 translocation (FITC stain) was visualized by immunofluorescence. DAPI, nuclear stain. Magnification: 400x. **(f)** HMEECs were transfected with NF-κB luciferase vector. Cells were pre-treated with curcumin (20 μM) for 1 h, followed by stimulated with NTHi for 5 h. NF-κB promoter activity was measured by luciferase assay. Data are mean ± s.d. (n = 3). (**b**,**f)** *p < 0.05, t-test. Displayed immunoblots are cropped images from full-length blots, presented in Supplementary Fig. S3. Data are representative of three or more independent experiments.

Next, we sought to determine the mechanism by which curcumin inhibits CXCL5 expression. Since activation of IKKβ-IκBα and p38 signaling pathways has been shown to be involved in NTHi-induced CXCL5 expression, we assessed the effect of curcumin on these pathways. Curcumin abrogated NTHi-induced IKKα/β phosphorylation (Fig. 6a). Moreover, curcumin inhibited IKKβ-CA-induced CXCL5 expression (Fig. 6b). Curcumin reduced NTHi-induced p38 phosphorylation (Fig. 6c). We also confirmed the inhibitory effect of curcumin on NTHi-induced p38 phosphorylation by immunofluorescence staining (Fig. 6d). Next, we determined the effect of curcumin on p65 nuclear translocation and NF-κB-driven luciferase promoter activity. Consistent with the above findings, curcumin suppressed NTHi-induced p65 nuclear translocation (Fig. 6e) and NF-κB luciferase activity (Fig. 6f). Thus, these data suggest that curcumin inhibits NTHi-induced CXCL5 expression via inhibition of both IKKβ-IκBα and p38 signaling pathways.

### Curcumin suppresses CXCL5 expression via up-regulation of negative regulator MKP-1

**Figure 7:**
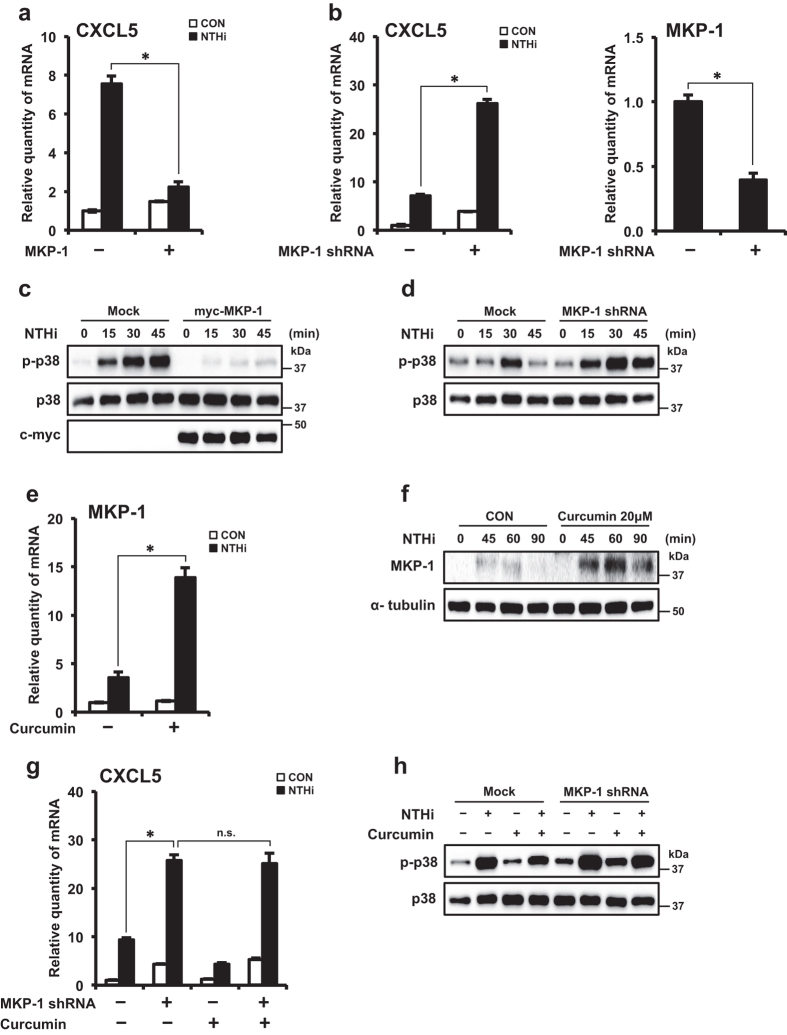
Curcumin suppresses NTHi-induced CXCL5 expression via up-regulation of negative regulator MKP-1. **(a,b)** HMEECs were transfected with (**a**) Mock or myc-MKP-1, (**b**) Mock or MKP-1 shRNA. Cells were stimulated with NTHi for 5 h, and CXCL5 mRNA expression was measured. Knockdown of MKP-1 protein by shRNA was confirmed by Q-PCR. **(c**,**d)** HMEECs were transfected with *(c)* Mock or myc-MKP-1 plasmid, *(d)* Mock or shMKP-1 shRNA. Cells were stimulated with NTHi for times indicated. Phospho-p38, total p38, MKP-1 protein levels were visualized by western blot. **(e)** HMEECs were pre-treated with curcumin (20 μM) for 1 h, followed by stimulation with NTHi for 1 h, and MKP-1 mRNA expression was measured. **(f)** HMEECs were pre-treated with curcumin (20 μM) for 1 h, followed by stimulation with NTHi for various time intervals as indicated in the figure. MKP-1 and α-tubulin protein levels were visualized by western blot. **(g)** HMEECs were transfected with Mock or MKP-1 shRNA. Cells were pre-treated with curcumin (20 μM) for 1 h, followed by NTHi stimulation for 5 h, and CXCL5 mRNA expression was measured. **(h)** HMEECs were transfected with Mock or MKP-1 shRNA. Cells were pre-treated with curcumin (20 μM) for 1 h, followed by NTHi stimulation for 30 min. Phospho-p38, total p38 protein levels were visualized by western blot. Data are mean ± s.d. (n = 3). (**a**,**b**,**e**) *p < 0.05, t-test. (**g)** *p < 0.05, ANOVA (Tukey’s post-hoc). n.s., not significant. Displayed immunoblots are cropped images from full-length blots, presented in Supplementary Fig. S4. Data are representative of three or more independent experiments.

MKP-1, a member of a class of dual specificity phosphatases collectively termed MAPK phosphatases, has been shown to be a key negative regulator of inflammatory responses via dephosphorylation and inactivation of MAPKs, including p38^35^,^36^. Since we identified the requirement of p38 MAPK activation in NTHi-induced CXCL5 expression, we determined the role of MKP-1 in CXCL5 regulation. Overexpression of MKP-1 suppressed NTHi-induced CXCL5 expression (Fig. 7a). Depletion of endogenous MKP-1 with MKP-1 shRNA enhanced CXCL5 mRNA expression (Fig. 7b). MKP-1 shRNA knockdown efficiency was confirmed by Q-PCR. These data suggest that MKP-1 is a negative regulator of NTHi-induced CXCL5 induction.

To further determine if MKP-1 acts as a negative regulator of CXCL5 induction via inactivation of p38, we evaluated the effect of MKP-1 on p38 phosphorylation. Overexpression of MKP-1 reduced NTHi-induced p38 phosphorylation (Fig. 7c). In contrast, depletion of MKP-1 enhanced p38 phosphorylation (Fig. 7d). These data suggest that MKP-1 negatively regulates NTHi-induced CXCL5 expression by targeting p38 MAPK.

Since curcumin and MKP-1 were identified to suppress activation of p38, we sought to determine if curcumin up-regulates MKP-1 expression. Curcumin markedly enhanced NTHi-induced MKP-1 expression at mRNA (Fig. 7e) and protein (Fig. 7f) levels. These data suggest the curcumin increases NTHi-induced expression of negative regulator MKP-1.

Having shown that curcumin inhibits NTHi-induced CXCL5 expression via inhibition of p38 and up-regulation of MKP-1 expression, we sought to determine if the inhibitory effect of curcumin on p38 is dependent on the up-regulation of MKP-1. Depletion of MKP-1 with shMKP-1 rendered curcumin treatment ineffective in inhibiting NTHi-induced CXCL5 expression (Fig. 7g). Additionally, curcumin no longer suppressed NTHi-induced p38 phosphorylation in the absence of MKP-1 (Fig. 7h). These data suggest that curcumin inhibits NTHi-induced activation of p38 via up-regulating MKP-1. Thus, our results suggest that curcumin inhibits NTHi-induced CXCL5 expression via MKP-1-dependent inhibition of p38 MAPK.

## Discussion

**Figure 8:**
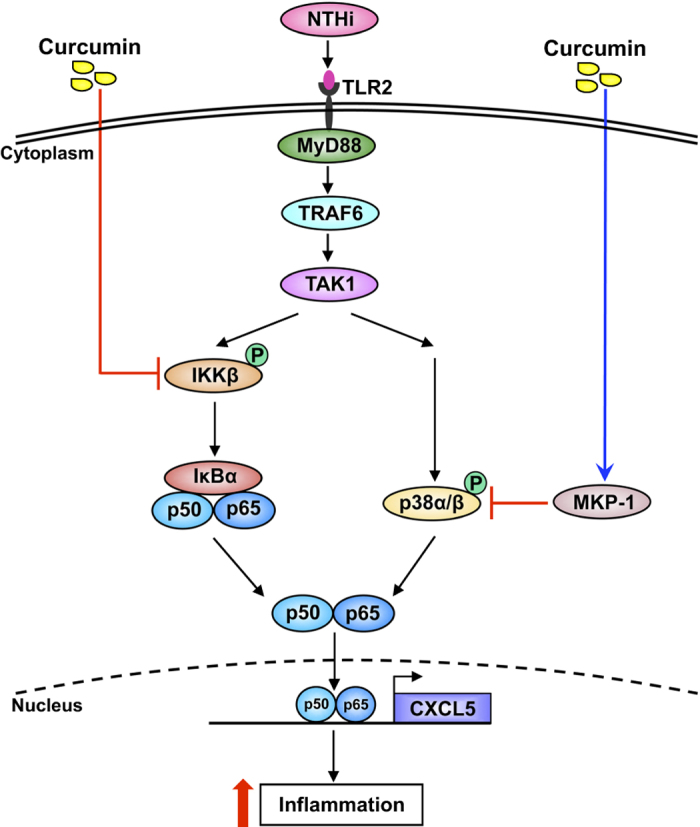
Schematic representation of NTHi-induced CXCL5 expression and curcumin-mediated suppression of CXCL5.

Inflammatory responses are essential for the containment, removal of the invading pathogens and recovery of the host. However, excess inflammation can be detrimental to the host as seen in OM^6^,^7^,^8^,^9^,^37^,^38^. Therefore, tight regulation of the intensity and duration of inflammatory responses is necessary. In the present study, we show that curcumin inhibits CXCL5 chemokine up-regulation in NTHi-induced OM model, *in vitro* and *in vivo*. We found that NTHi up-regulated CXCL5 expression by activating IKKβ-IκBα and p38 MAPK pathways via NF-κB nuclear translocation-dependent and -independent mechanism. Curcumin not only inhibited the positive IKKβ pathway but also up-regulated the expression of MKP-1, a key negative regulator of p38 MAPK, thereby suppressing CXCL5 expression by dual action. Thus, the current study provides novel insights into the molecular mechanism underlying the tight regulation of neutrophil attractant chemokine CXCL5 in the pathogenesis of NTHi-induced OM and also demonstrates the potential of curcumin as a novel therapeutic for treating OM (Fig. 8).

NF-κB was found to be the major transcription factor regulating NTHi-induced CXCL5 expression. In our study, both IKKβ-IκBα and p38 MAPK pathways were found to act via p65 subunit to regulate NF-κB transcriptional activity, albeit through different mechanisms to induce CXCL5 expression. Under resting conditions NF-κB is present in the cytoplasm bound to IκBα. Upon activation of upstream signaling pathways, NF-κB dissociates from IκBα and translocates to the nucleus to regulate gene expression. CAPE, an inhibitor of NF-κB nuclear translocation, inhibited p65 nuclear translocation and suppressed NF-κB transcriptional activity that in turn suppressed CXCL5 expression. Interestingly p38 MAPK inhibitor SB203580 failed to inhibit p65 nuclear translocation but suppressed NF-κB transcriptional activity and CXCL5 expression. These findings suggest that p38 MAPK regulates NF-κB transcriptional activity itself but not p65 nuclear translocation. Previous studies have reported that post-translational modifications such as phosphorylation and acetylation of p65 are critical for promoting its DNA binding and interaction with transcriptional machinery to regulate gene expression. p38 MAPK was found to regulate the acetylation status of p65 but not its phosphorylation. In response to activating stimuli, p38 was found to phosphorylate the transcriptional coactivator p300 (a histone acetyltransferase). Phosphorylated p300 binds to and acetylates K310 residue on p65. Acetylation of K310 was shown to enhance p65 transcriptional activity^39^. Therefore it likely that p38 MAPK mediates NTHi-induced CXCL5 expression in a similar manner by increasing NF-κB transcriptional activity, independent of p65 nuclear translocation.

OM, a leading cause of conductive hearing loss in children, is caused by NTHi^1^. OM is characterized by the presence of excessive inflammation in the middle ear^3^,^40^. Current therapies for OM involve the use of analgesics and antipyretics for symptomatic treatment^41^. Though these medications are effective during certain stages of the disease, prolonged usage poses the risk of serious side effects due to unknown “off-targets” and weakened immune system. Decongestants, antihistamines, and corticosteroids have not been effective in treating OM^42^. Prophylactic use of antibiotics has rendered over 80% of the NTHi strains drug-resistant^4^,^5^. Also, development of vaccines against NTHi remains a challenge due to the high genetic diversity of NTHi strains and high antigenic variability of surface-exposed antigens^43^,^44^. Thus, there is an urgent need for developing alternative therapeutics for OM with increased efficiency and safety. Therefore, identifying the underlying molecular mechanisms leading to up-regulation of inflammation is critical for the development of novel therapeutic strategies with increased specificity and reduced side effects. Interestingly, in the current study we provide evidence that curcumin inhibits NTHi-induced CXCL5 chemokine expression in middle ear epithelial cells. Interestingly, both pre-infection and post-infection treatment with curcumin not only inhibited NTHi-induced CXCL5 up-regulation but also suppressed PMN infiltration into the middle ear in a mouse model of OM. Curcumin treatment’s efficacy in inhibiting CXCL5 expression and PMN recruitment post-NTHi infection is of particular clinical significance. Recently chemokines and chemokine receptors are increasingly considered as targets for developing new drugs to control inflammation^45^. Our finding that curcumin suppresses NTHi-induced CXCL5 chemokine expression is of particular relevance in the current scheme of identifying chemokine-drug combinations to treat inflammation. Thus, curcumin could be repurposed as a new therapeutic for treating OM.

Curcumin is a nutraceutical that has been in use in South Asian countries for many centuries owing to its medicinal properties^20^. Curcumin can interact with a myriad of signaling molecules including transcription factors, protein kinases, growth factors, receptors, adhesion molecules, pro-inflammatory cytokines^46^, thus explaining it pleiotropic therapeutic potential against a wide range of diseases. Curcumin does not present a dose-limiting toxicity, making it suitable for prolonged usage. Completed clinical trials reported usage of curcumin dosage ranging from 0.045 to 8 g/day. Currently, 38 clinical trials evaluating the efficacy of curcumin at a dosage ranging from 0.18 to 8 g/day for treating pathologies such as Alzheimer’s disease, diabetes, kidney disease, Crohn’s disease, cancer are underway^47^. United States Food and Drug Administration classified curcumin as GRAS (generally recognized as safe), warranting its use as a supplement. In the current study, we identified a novel role of curcumin in suppressing CXCL5 chemokine production. Co-administration of curcumin along with piperine, docetaxel, soy isoflavones, bioperine, lactoferrin, mesalamine in clinical trials^48^,^49^,^50^,^51^,^52^, suggest the possibility of customizing curcumin-based therapies to maximize its therapeutic efficiency. Since bioavailability of curcumin is a challenge^47^, further studies combining the use of adjuvants, lipids, nanoparticles are needed to elucidate further the potency of curcumin in treating OM.

Another relevant finding of biological significance in the current study is the dual acting mechanism of curcumin in inhibiting CXCL5 expression. Curcumin inhibited NTHi-induced IKKβ phosphorylation, thereby suppressing CXCL5 up-regulation. Moreover, we found that curcumin also inhibits NTHi-induced CXCL5 expression via MKP-1-dependent suppression of p38 MAPK. In the absence of MKP-1, curcumin failed to suppress CXCL5 expression. Due to the importance of p38 in maintaining homeostasis, up-regulation of negative regulator MKP-1 by curcumin could play an important role in controlling the over-active immune responses with minimal side effects. This finding is of particular translational significance due to the attractiveness of targeting overactive inflammation via induction of negative-regulators^19^.

We previously demonstrated that dexamethasone glucocorticoid inhibits p38 MAPK via up-regulation of MKP-1^53^. Glucocorticoids owing to their potent immunosuppressive and anti-inflammatory effects have been in use for treating a gamut of diseases such as asthma, allergies, skin disorders, multiple sclerosis, immune disorders and cancer. However, prolonged usage has been reported to cause severe, sometimes irreversible side effects such as osteoporosis, endocrine and metabolic disorders, behavioral and cognitive changes, gastrointestinal tract complications, uveitis and weakened immune system^54^. Numerous studies over the past years have demonstrated that curcumin’s efficacy in resolving pathologies was similar to that of dexamethasone^55^,^56^. No evidence of side effects with low to moderate consumption of curcumin exists. With a higher curcumin dosage 12 g/day, mild symptoms such as diarrhea, low blood sugar, abdominal pain, and indigestion have been reported^47^. Additionally, curcumin has been reported to aid in overcoming the side effects of glucocorticoid usage^57^. Curcumin is also effective against oxidative stress, characteristic of many inflammatory conditions. Curcumin supplementation could be an effective disease preventive strategy due to its immunomodulatory activity^58^. Thus, curcumin fits the bill for an alternative therapeutic with minimal side effects.

In conclusion, our study demonstrates for the first time that curcumin is a potent inhibitor of CXCL5 chemokine, which could, in turn, suppress inflammation. Further studies promoting curcumin bioavailability may provide means to develop therapies to modulate inflammation more stringently without adverse effects. Development of drug delivery systems in the form of a topical ointment and ear drops could be of clinical significance in treating OM. The findings of this study may have applications in a broader context to other pathologies including chronic obstructive pulmonary disease, tuberculosis, cancer and Alzheimer’s disease.

## Materials and Methods

### Reagents and antibodies

IKKβ inhibitor IV and MG-132 were purchased from EMD Millipore. CAPE, SB203580 were purchased from Enzo Life Sciences. Curcumin was purchased from Sigma. Antibodies for p-IKKα/β (#2697), p-p38 (#9211), p38 (#9212), anti-rabbit HRP-linked antibody (#7074) and anti-mouse HRP-linked antibody (#7076) were purchased from Cell Signaling Technology. Antibodies for IKKα/β (sc-7607), p65 (sc-8008), MKP-1 (sc-370), α-tubulin (sc-69969), c-Myc (sc-40), anti-mouse FITC-conjugated antibody (sc-2010), anti-rabbit Rhodamine-conjugated antibody (sc-2091) were purchased from Santa Cruz Biotechnology.

### Cell culture

All media described below were supplemented with 10% fetal bovine serum and 100 U/ml penicillin and 100 μg/ml streptomycin (Gibco). Human middle ear epithelial cells (HMEECs) were maintained in DMEM (Cellgro) supplemented with BEGM SingleQuots (Lonza). Lung epithelial A549 cells were maintained in F-12K medium (Gibco). Human cervical epithelial HeLa cells were maintained in DMEM (Cellgro). Cells were cultured at 37 °C in a humidified 5% CO_2_ atmosphere.

### Bacterial strains and culture conditions

Clinical isolates of NTHi strains 12, 2627, 9274 were used for this study^28^,^29^. NTHi was grown on chocolate agar plate in 5% CO_2_ atmosphere for 16 h, followed by overnight culture in brain heart infusion (BHI) broth supplemented with 3.5 μg/ml NAD and 10 μg/ml hemoglobin (BD Biosciences). Subsequently, bacteria were subcultured in 5 ml fresh BHI broth and the growth was monitored by measurement of optical density (OD). Log phase bacteria were harvested, washed and re-suspended in DMEM for *in vitro* experiments and isotonic saline for *in vivo* experiments. For all *in vitro* experiments the cells were stimulated with NTHi at a multiplicity of infection (MOI) of 50, with an exception for dose-dependent experiment. Cells were stimulated with NTHi for 5 h, or otherwise as indicated. For inhibition study, cells were pretreated with the respective inhibitor for 1 h prior to NTHi stimulation. For post-treatment studies cells were treated with curcumin 1 h after NTHi stimulation.

### Plasmids, transfection and luciferase assay

The expression plasmids, for dominant negative (DN) forms of TLR2, TLR4, TRAF6, MyD88, IKKα (K44M), IKKβ (K49A), p38α (fp38α (AF)), p38β (fp38β2 (AF)), trans dominant IκBα (S32A/S36A), constitutively active form of IKKβ (IKKβ-CA, S176E/S180E), p65 have been described previously^31^,^59^. NF-κB luciferase reporter vector (pGL4.32) was purchased from Promega. Myc-MKP-1 overexpression plasmid was subcloned from previously described pSG5–MKP-1 plasmid^53^. MKP-1 sequence was amplified using the primers 5′-GGTCTCGAGCGATGGTCATGGAAGTGG-3′ and 5′-GGTGGATCCTCCGCAGCTGGGAGAGGT-3′ and inserted into the XhoI and BamHI sites of pcDNA3.1/*myc*His(−) vector. All transient transfections were performed using TransIT-LT-2020 transfection reagent (Mirus) according to the manufacturer’s protocol. Cells were assayed 48 h after transfection. Empty vector was transfected as a control. pSV-β-Galactosidase vector was used as a control for luciferase assay. Luciferase activity and β-Galactosidase activity was measured using Luciferase Assay System (Promega) and β-Galactosidase Enzyme Assay system (Promega). NF-κB luciferase activity was normalized with respect to β-Galactosidase activity.

### RNA-mediated interference

Human siRNA (Control, D001810-10; IKKβ, L003503-00; p65, L003533-00; TAK1, M003790-06-0005) were purchased from GE Health care. Cells were transfected with 20 nM siRNA using DharmaFECT-4 (Thermo Scientific) according to manufacturer’s protocol. Human pSUPER-shMKP-1 knockdown construct was previously described^60^. shMKP-1 was transfected using TransIT-LT-2020 transfection reagent (Mirus) according to manufacturer’s protocol. Cells were assayed 48 h after transfection.

### Real-time quantitative PCR (Q-PCR) analysis

Total RNA was isolated with TRIzol reagent (Life Technologies) according to manufacturer’s protocol. Reverse transcription reaction was performed with 1 μg RNA using TaqMan reverse transcription reagents (Applied Biosystems) according to manufacturer’s protocol. Quantitative PCR was performed using Fast SYBR Green Master Mix. PCR reactions containing 2X universal master mix, 1 μL template cDNA, 500 nM primers in a final volume of 12.5 μL, were amplified and quantified using StepOnePlus Real-Time PCR system (Applied Biosystems). Relative quantities of mRNAs were obtained using the comparative Ct method and were normalized to human Cyclophilin or mouse glyceraldehyde-3-phosphate (GAPDH); serving as an endogenous control. Human (h) and mouse (m) primer sequences are as follow: hCXCL5 5′-GTGGTAGCCTCCCTGAAGAAC-3′ and 5′-TCCTTGTTTCCACCGTCCAA-3′; hTAK1 5′-GCAACAGAGTGAATCTGGAC-3′ and 5′-CAGACATGTCAGCACTCATC-3′; mCXCL5 5′-GCTGGCATTTCTGTTGCTGTTC-3′and 5′-GGCAGCTTCAGCTAGATGCT-3′. hMKP-1, hCyclophilin and mGAPDH primer sequences were previously described^60^.

### Enzyme-linked immunosorbent assay (ELISA)

Cells were stimulated with NTHi for 12 h. Culture media was harvested and centrifuged at 12,000 × *g* for 10 min to precipitate cell debris. Culture supernatants were assayed using human ENA78/ CXCL5 ELISA kit (Sigma) according to manufacturer’s protocol. OD was measured using Benchmark Plus microplate spectrophotometer. A standard curve showing the relationship between concentration and OD was generated for CXCL5 protein standards. CXCL5 protein concentration in culture supernatants was determined by interpolating from the standard curve.

### Western Blot Analysis

Following NTHi stimulation, whole cell extracts were recovered with lysis buffer containing 50 mM Tris-HCl (pH 7.4), 1% Nonidet P-40, 0.25% deoxycholate, 150 mM NaCl, 1 mM EDTA, 1 mM NaF, supplemented with1 mM PMSF, 1 mM Na_3_VO_4_ and protease inhibitor cocktail). Cell extracts were incubated on ice for 30 min and centrifuged at 12,000 × *g* for 30 min to precipitate cell debris. Supernatants were separated on 10% SDS-PAGE gel, transferred to polyvinylidene difluoride (PVDF) membrane. The membrane was blocked with blocking buffer (TBS containing 0.1% Tween 20 (TBS-T) and 5% nonfat dry milk). After 3 washes with TBS-T, the membrane was incubated overnight with primary antibodies at 1: 1,000–1: 2,000 dilutions in antibody dilution buffer (TBS-T containing 5% BSA) at 4 °C. After 3 washes with TBS-T, the membrane was incubated with corresponding secondary antibody at 1: 5,000 dilution in blocking buffer for 1 h. After 3 washes with TBS-T, the proteins were visualized using Amersham ECL Prime Detection Reagent (GE Healthcare). Images have been cropped for presentation. Full-size images are presented in Supplementary Figs 1–4.

### Mice and Animal Experiments

C57BL/6 mice were purchased from Jackson Laboratories. Anesthetized mice were trans-tympanically inoculated with NTHi at a concentration of 5 × 10^7^ CFU per mouse. Saline was inoculated as control. For inhibition studies, mice were injected intraperitoneally (i.p) with curcumin (50 mg/kg) 1 h prior or 1 h after NTHi inoculation. For mRNA analysis, mice were sacrificed 6 h post-NTHi inoculation. Total RNA was extracted from the dissected mice middle ear. For PMN analysis, mice were sacrificed 9 h post-NTHi inoculation. Middle ear effusions from mice were harvested with 10 μl saline (x3). Following cytocentrifugation cells were stained with Diff-Quik stain kit (Siemens) according to manufacturer’s protocol. Images were recorded with light microscopy system (AxioVert 40 CFL, AxioCam MRC and AxioVision LE Image system, Carl Zeiss). All animal studies were carried out in accordance with the guidelines of, and were approved by, The Institutional Animal Care and Use Committee at Georgia State University.

### Immunofluorescence staining

HMEECs were grown on 18 mm round glass coverslips (VWR). Cells were fixed in 4% paraformaldehyde. Cells were then incubated with primary antibodies at 1: 100–1: 400 dilutions. Primary antibody was detected with FITC or Rhodamine-conjugated secondary antibody. The coverslips were mounted onto glass slides using VECTASHIELD HardSet Antifade mounting Medium with DAPI (Vector). Images were recorded with fluorescence microscopy system (AxioVert 40 CFL, AxioCam MRC and AxioVision LE Image system, Carl Zeiss).

### Statistical analysis

All experiments were repeated in at least three independent experiments. Data are shown as mean ± standard deviation (s.d.). The difference in means was assessed with unpaired student’s t-test for data with two conditions (*k* = 2), ANOVA (with Tukey’s post-hoc) for data with more than two conditions (*k* > 2), using SPSS 22 statistics software (IBM). *p < 0.05 was considered statistically significant.

## Additional Information

**How to cite this article**: Konduru, A. S. *et al*. Curcumin suppresses NTHi-induced CXCL5 expression via inhibition of positive IKKβ pathway and up-regulation of negative MKP-1 pathway. *Sci. Rep.* **6**, 31695; doi: 10.1038/srep31695 (2016).

## Supplementary Material

Supplementary Information
