# Supplementary material for: Curcumin suppresses NTHi-induced CXCL5 expression via inhibition of positive IKKβ pathway and up-regulation of negative MKP-1 pathway
Source: Sci Rep. 2016 Aug 19;6:31695. doi: 10.1038/srep31695 (PMC4990917; doi:10.1038/srep31695)
Supplement: Supplementary Information [file srep31695-s1.pdf]

Supplementary Information

Curcumin suppresses NTHi-induced CXCL5 expression via inhibition of positive IKKβ pathway and up-regulation of negative MKP-1 pathway

Anuhya S Konduru, Byung-Cheol Lee, Jian-Dong Li

Fig. 3a

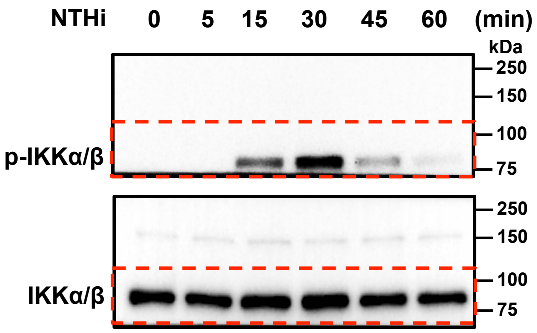

Fig. 3d

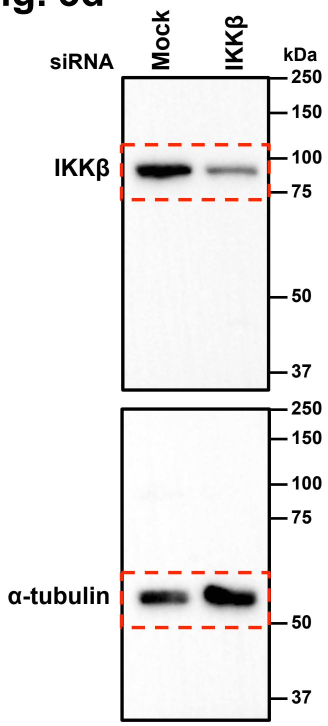

Fig. 3h

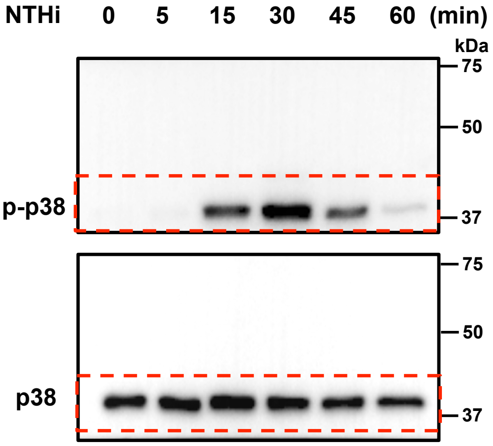

Supplementary Figure S1. Uncropped Immunoblot images with molecular weight markers shown in Fig. 3

Fig. 4e

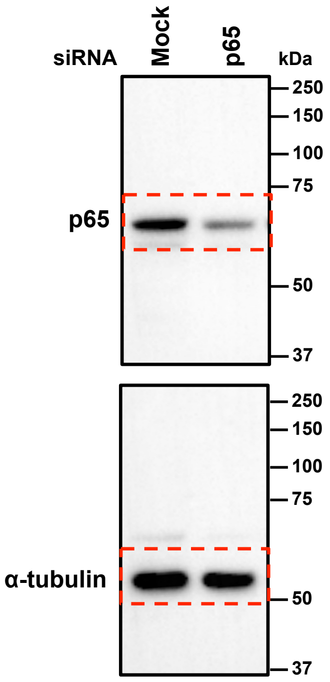

Supplementary Figure S2. Uncropped Immunoblot images with molecular weight markers shown in Fig. 4

Fig 6a

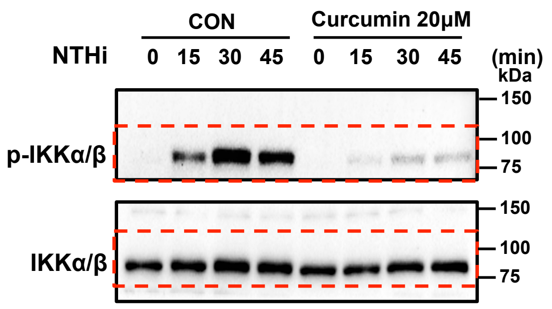

Fig 6c

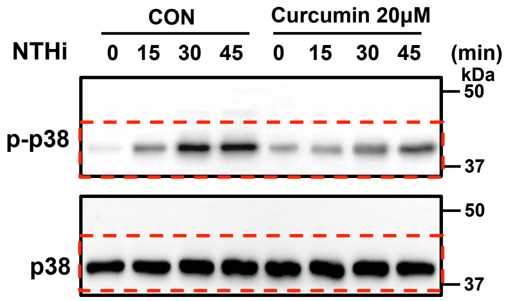

Supplementary Figure S3. Uncropped Immunoblot images with molecular weight markers shown in Fig. 6

Fig 7c

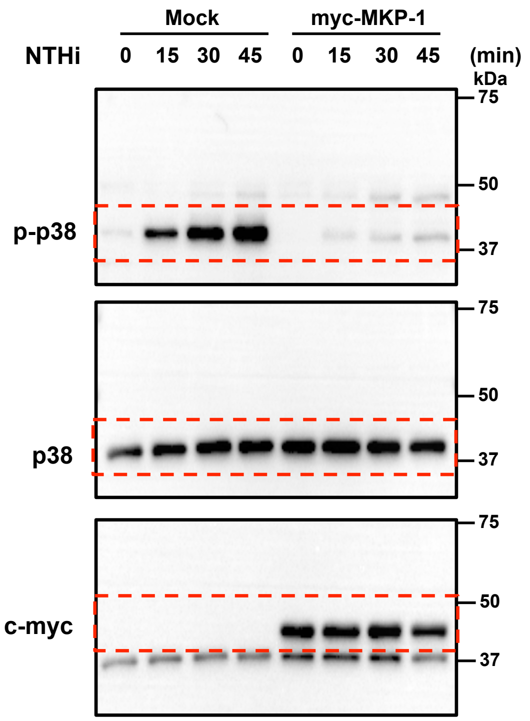

Fig 7d

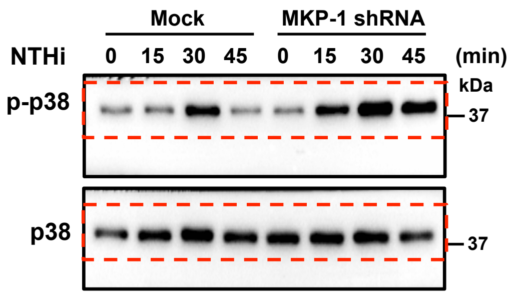

Fig 7f

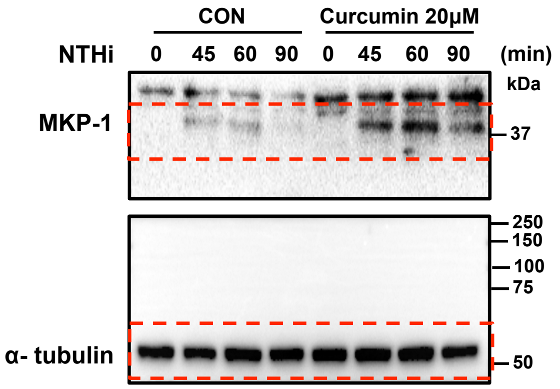

Fig 7h

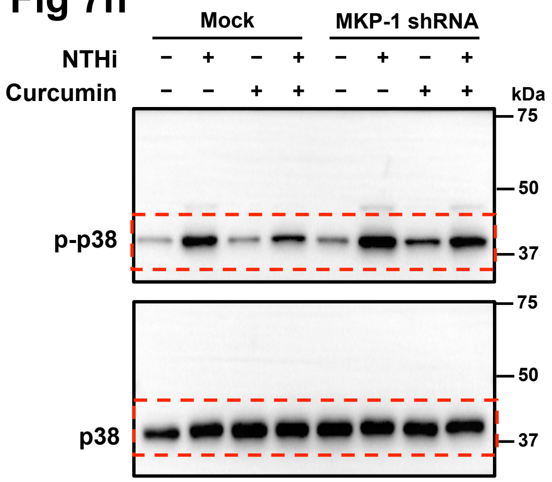

Supplementary Figure S4. Uncropped Immunoblot images with molecular weight markers shown in Fig. 7
